# Review of Machine Learning Methods for the Prediction and Reconstruction of Metabolic Pathways

**DOI:** 10.3389/fmolb.2021.634141

**Published:** 2021-06-17

**Authors:** Hayat Ali Shah, Juan Liu, Zhihui Yang, Jing Feng

**Affiliations:** Institute of Artificial Intelligence, School of Computer Science, Wuhan University, Wuhan, China

**Keywords:** machine learning, prediction, metabolic pathway, enzymes, biochemical reaction, substrate, metabolites

## Abstract

Prediction and reconstruction of metabolic pathways play significant roles in many fields such as genetic engineering, metabolic engineering, drug discovery, and are becoming the most active research topics in synthetic biology. With the increase of related data and with the development of machine learning techniques, there have many machine leaning based methods been proposed for prediction or reconstruction of metabolic pathways. Machine learning techniques are showing state-of-the-art performance to handle the rapidly increasing volume of data in synthetic biology. To support researchers in this field, we briefly review the research progress of metabolic pathway reconstruction and prediction based on machine learning. Some challenging issues in the reconstruction of metabolic pathways are also discussed in this paper.

## Introduction

Metabolic pathways are a series of enzymatic reactions in a cell, where the products of reactions are the substrates for subsequent reactions. The reactants, products, and intermediates of an enzymatic reaction are known as metabolites. There are many metabolic pathways have been identified out and been stored and characterized in several public repositories according to their functions, including KEGG (Ogata et al., 1998; Ogata et al., 1999; Okuda et al., 2008; Kanehisa et al., 2019), MetaCyc (Karp 2002b; Caspi 2006; Caspi et al., 2008; Caspi et al., 2018), BioCyc (Karp et al., 2019). However, there are still many metabolic pathways remain uncharacterized, because some components of them are not identified (Roche-Lima 2016). The reconstruction of metabolic pathways aims to refine incomplete pathways caused by the lack of enzymes, reactions or relationships between reactions. Some researchers reconstruct the metabolic pathways of an organism based on reference pathways. That is, mapping the incomplete pathways onto the reference ones to identify the unknown parts. A variety of reference-based approaches have been developed to reconstruct the metabolic pathways, including BlastKOALA (Kanehisa et al., 2016), KAAS (Moriya et al., 2007), GhostKOALA (Kanehisa et al., 2016), and RAST (Aziz et al., 2008). Now that there are many metabolic pathways have been collected and organized in some public databases, such as KEGG (Ogata et al., 1998; Ogata et al., 1999; Okuda et al., 2008; Kanehisa et al., 2019), MetaCyc (Karp 2002b; Caspi 2006; Caspi et al., 2008; Caspi et al., 2018), BioCyc (Karp et al., 2019), Brenda (Schomburg 2002; Jeske et al., 2019), Rhea (Lombardot et al., 2019), and EcoCyc (Karp 2002a), the reference-based methods make use of the pathways in the public databases as references, and map the protein sequences of an organism onto the reference pathways according to sequence homology (Herrgård et al., 2008) to reconstruct the metabolic pathways of the organism. However, if some enzymes or reactions are also missed in reference pathways, such reference-based methods may reconstruct incorrect metabolic pathways and lead to incorrect elucidation. Furthermore, such kind of methods cannot predict new reactions or enzymes that do not exist in the reference pathways. Other researchers reconstruct metabolic pathways by beginning with predicting gene sequences from genome data using gene markers (Besemer 2001). The predicted gene sequences are first assigned initial functions by a variety of computational approaches such as clustering, similarity calculation with known sequences, and so on. Then they are “attached” to pathways by choosing templates from metabolic pathway database which best incorporate all observed functions (Overbeek 2000; Mascher et al., 2019); then a basic functional model is created and evaluated against known data. Such kind of methods depends on the deduced gene sequence; however, the protein translated from coding sequences may be incorrect due to the problem of frameshift, resulting wrong pathways. For eukaryote, prediction of gene sequences is even more difficult due to the existence of introns.

In order to overcome the shortcomings of above methods, it is necessary to have strong evidence on genome context association, such as gene-gene interactions (Gurkun, 2012), classification and clustering based on their function and phylogenetic profiling (Sithambranathan et al., 2020). Now that machine learning has outstanding ability in dealing with large and complex data sets and a large amount of data have been obtained through large projects, it is an inevitable trend to apply machine learning to the reconstruction of metabolic pathways. Over the past decade, there have been many researches focusing on the modeling and reconstruction of metabolic pathways. Wang et al. (2017) have surveyed some computational tools for design and reconstruction of metabolic pathways. Cuperlovic-Culf (2018) has reviewed related work on modeling of metabolic pathways based on machine learning techniques. Kim et al. (2020) have summarized the machine learning applications in systems metabolic engineering. However, there is lack of review on machine learning applications on predicting components in metabolic pathways. In this paper, we briefly review the machine learning approaches for the predictions of metabolic pathways and their components, including enzymes, metabolites, and reactions. This review, together with other reviews, can provide more comprehensive knowledge for machine learning algorithms in the prediction and reconstruction of the metabolic pathways.

The remainder of this paper is organized as follows: *Prediction or Reconstruction of Metabolic Pathways* describes the prediction and reconstruction of the metabolic pathways. *Prediction of Missing Enzymes* presents the prediction of missing enzymes. *Identification of Metabolites* introduces machine learning methods for predicting metabolites, followed by *Prediction of Reactions*, which describes prediction of reactions. *Conclusion* concludes this paper.

## Prediction or Reconstruction of Metabolic Pathways

A metabolic pathway is a linked series of chemical reactions that occur within a cell. These reactions are catalyzed by enzymes, where the product of one enzyme acts as the substrate for the next. The reactants, products, and intermediates of an enzymatic reaction are known as metabolites. In a pathway, the initial chemical (metabolite) is modified by a sequence of enzymatic reactions.

There are three pipelines of computational methods for analyzing metabolic pathways: prediction (Bagheri et al., 2019; Faust et al., 2011), design or reconstruction (Qi et al., 2014), and optimization (Ebenhöh and Heinrich 2001; Planes and Beasley 2009; Jeanne et al., 2016). The pipeline of prediction of metabolic pathways is to predict the metabolic pathways that a given molecular belongs to, which can help to understand the metabolic mechanism of the molecular. For example, in drug discovery, predicting the metabolic pathway of a drug compound involving in is very useful for knowing how the drug is absorbed, distributed, metabolized, and excreted. The purpose of the metabolic pathway design or reconstruction is to design or find the routines of enzymatic reactions that convert one metabolite (source) to the others (products). Reconstruction of metabolic pathways is also useful for finding functional modules or building the metabolic network of an unknown organism. In metabolic engineering, design or reconstruction of the metabolic pathways to a specific product can help to modify a microbial strain to enable and strengthen the new pathways for efficient production of biochemical. The optimization of metabolic pathways involves in finding or generating the optimal pathways based on the predetermined criteria, such as maximizing production yield of target products, minimizing the number of reactions, and so on. The optimization of metabolic pathways usually needs to meet some constraints, for example, with specific enzymes and with the highest yield of target products. Therefore, constraint-based methods are usually used, and in most cases additional metabolic flux analysis data is needed for the optimization of pathways, which is out of the scope of this review.

### Prediction of Metabolic Pathways

Now that the annotated metabolic pathways been organized into different categories according to their functions. For a new or unknown molecular, knowing which or what kind of pathways it belongs to can help to understand its metabolic mechanism, which is very useful for drug discovery. Therefore, the metabolic pathways prediction mentioned in this paper refers to identifying the metabolic pathways that a compound involves in. There have some machine learning methods been applied to building prediction models for pathways. For example, Baranwal et al. (2019) proposed a hybrid framework of random forest (RF) and a graph convolution neural network for predicting the classes of metabolic pathways that a compound belongs to. Their method can only identify metabolic pathway types of compounds rather than the actual metabolic pathways. There remains a gap between predicting the type of metabolic pathways and predicting actual metabolic pathways to which the compound belongs. To fill this gap, Jia et al. (2020) proposed a similarity-based model for predicting the metabolic pathways of given compounds. They regarded every pair of compound and metabolic pathway as a sample, and represented each sample by seven features extracted from seven associations of compounds. And then they built a binary classification model with the RF algorithm to output “yes” or “no” for every pair, where “yes” means the compound belongs to the pathway, and “no” for not. However, the method is only suitable for known pathways, and it is impossible to predict whether the compounds belong to unknown pathways. Moreover, just predicting metabolic pathways that given compounds belong to is not enough to fully understand their roles in the metabolism, and thus it is necessary to reconstruct or design the metabolic pathways involved by the compounds.

## Reconstruction of Metabolic Pathways

The reconstruction of a metabolic pathway connects metabolites and pairs of biochemical reactions catalyzed by enzymes, marking the routes and connecting source molecules to target molecules. Pathway reconstruction can be either knowledge-driven objective (KDO) or data-driven objective (DDO) (Viswanathan et al., 2008). Since knowledge-driven pathway construction incorporates a large amount of domain knowledge, the development of a detailed pathway knowledge base for particular domains of interest, such as a cell type, disease, or system is needed. Such knowledge base serves as the pathway resources that help to reliably identify and extract the pertinent entities and interactions. For example, Karp and his collaborators developed a pathway software, Pathologic, to reconstruct metabolic pathways using functional annotations onto the MetaCyc collection or reactions of pathways (Karp et al., 1999; Paley and Karp 2002). However, the development of domain knowledge is a tedious task. Data-driven pathway construction is used to generate relationship information of genes or proteins identified in a specific experiment. Different from KDO, DDO starts from genes or proteins whose relationships are not well understood. In order to identify the relationship of the genes or proteins, reference-based or template-based methods based on mapping a group of gene and protein sequences of an organism to known reference pathways have been commonly adopted (Overbeek 2000; Herrgård et al., 2008; Mascher et al., 2019). However, they generally cannot predict new reactions that do not exist in a reference pathway. Some researchers proposed ab initio methods that do not use reference pathways to reconstruct metabolic pathways. Most of these methods employ probabilistic inference methods such as graphical models and Bayesian networks (Jansen et al., 2003; Friedman 2004; Werhli et al., 2006; Zhao et al., 2012) or ordinary differential equations (ODEs) (Koza et al., 2001; Schmidt et al., 2011). Ab initio reconstruction methods can predict novel reactions and interactions, but their accuracies tend to be low leading to a lot of false positives. In order to address the limitations of reference-based and ab initio methods, Qi et al. (2014) proposed to combine existing pathway knowledge and a Bayesian probabilistic graphical model together, and thus to improve both the coverage and accuracy of metabolic pathway construction. However, the pathway built through this method may be an incomplete elucidation due to the unknown enzyme genes. Therefore, besides inferring interactions or reactions, predicting the composition of the pathway from a reference database for the organism is necessary for pathway reconstruction.

### Design of Metabolic Pathways

In metabolic engineering, one usually needs to design or find metabolic pathways to chemicals of interest that meets certain constraints in a strain from living organisms. In order to expand the chemical repertoire for the production of compounds, a major effort is required in the development of novel design tools that target chemical diversity through rapid and predictable protocols. Addressing that goal involves retrosynthesis approaches that explore the chemical biosynthetic space. The basic idea of a retrosynthesis approach is to iteratively break down a target molecule into simpler molecules that can be combined chemically or enzymatically to produce it until all required compounds are either commercially available or present in the microbial strain of choice (Koch et al., 2020). Several researchers have reviewed efforts of retrosynthesis (Planson et al., 2012; Wang et al., 2017; Lin et al., 2019). However, the complexity associated with the large combinatorial retrosynthesis design space has often been recognized as the main challenge hindering the approach (Delépine et al., 2018). Pathway pruning methods (Gerlee et al., 2009) or optimization-based (Küken and Nikoloski 2019; Koch et al. 2020) methods are usually used to explore the chemical biosynthetic space. For example, Connor et al. (2017) proposed a Retrosynthesis approach Based on Molecular Similarity; Delépine et al. (2018) developed an automated open source workflow for retrosynthesis based on generalized reaction rules that perform the retrosynthesis search from chassis to target through an efficient and well-controlled protocol; Koch et al. (2020) proposed to explore the bioretrosynthesis space using the Monte Carlo Tree Search reinforcement learning method, guided by chemical similarity. However, the integration of both metabolic engineers’ expertise and years of lessons from the industry is not enough when performing pathway searching and ranking, resulting that the designed pathway may be far from the optimal.

### Issues Need to Be Addressed

In order for the reconstruction of metabolic pathways, *de novo* reaction prediction is still a significant challenge. Though some methods can learn the enzymatic reaction likeness to predict whether a compound-compound pair is possible converted by an enzymatic reaction, and even can find hidden reactions among many compounds at a time, they are insufficient to predict a multistep metabolic pathway correctly.

In order to construct the metabolic pathways, more efforts should be paid for the difficulties of distinguishing unidentified parts of the pathways and structuring pathways for desired products. In particular, the extraction of useful information from metabolomics is necessary to structure the pathways. Moreover, the computational algorithms should consider the case that an enzyme connects with at least two substrates at the same time to increase the yield of production. Though the graph-based approach can be used to analyze flux-balanced pathways in the metabolic network (Arabzadeh et al., 2018), it usually needs extra post-processing steps to adjust co-metabolites of the predicted pathway that could be unbalanced. In addition, the prediction of catalytic activities of enzymes has become one of the hot research topics.

## Prediction of Missing Enzymes

### Description of the Problem

An enzyme is a protein catalyst that acts on substrates and converts them into molecules known as products. If a particular function is not assigned to a protein, any reaction catalyzed by that protein will be referred to as a missing enzyme or pathway hole (Green and Karp, 2004). The missing enzymes make it difficult to understand the behaviors of them in the metabolic pathways. The comprehensive and accurate reconstruction of the metabolic pathways in an organism includes the identification of the missing enzymes catalyzing the reactions of the pathways. Basically, identification of missing enzymes contains two steps: selecting candidates and evaluating candidates. The selection of candidates is to find a set of proteins or encoding genes that may catalyze the specific reaction based on some strategies, such as calculating similarities, finding correlations, and so on; and the evaluation of the candidates is to identify the missing enzyme catalyzing the reaction from the candidates to fill in the pathway hole.

### Identification of Candidates of Missing Enzymes

Traditional computational efforts to identify missing enzymes in metabolic pathways have focused on finding candidate enzymes based on sequence homology (Green and Karp, 2004). That is, calculating the similarity of a sequence from the organism of interest to sequences that catalyze the same reaction of other organisms with known metabolic pathways. However, such sequence homology methods fail to identify enzymes encoded by genes with poor sequence homology to known metabolic enzymes. To solve the problem, Green and Karp (2004) developed a method that efficiently combined homology and pathway-based evidence to identify candidates; Yamanishi et al. (2007) used supervised network inference to select enzyme encoding gene candidates based on the estimation of the functional association between the genes with respect to chromosomal proximity and evolutionary association; Kharchenko et al. (2006) showed that a number of different types of functional association evidence, including phylogenetic profile co-occurrence, physical clustering of genes on the chromosome and protein interaction data can be used to identify metabolic enzyme encoding genes, and presented two kinds of integration methods, that is, direct likelihood-ratio (DLR) method and alternating decision trees (ADT) built by Adaboost. Since such kind of methods is based on the generally accepted biological hypothesis to build the models, the obtained candidates can more likely fill the pathway hole. However, complicated strategies are usually needed to integrate knowledge into the models.

**FIGURE 1:**
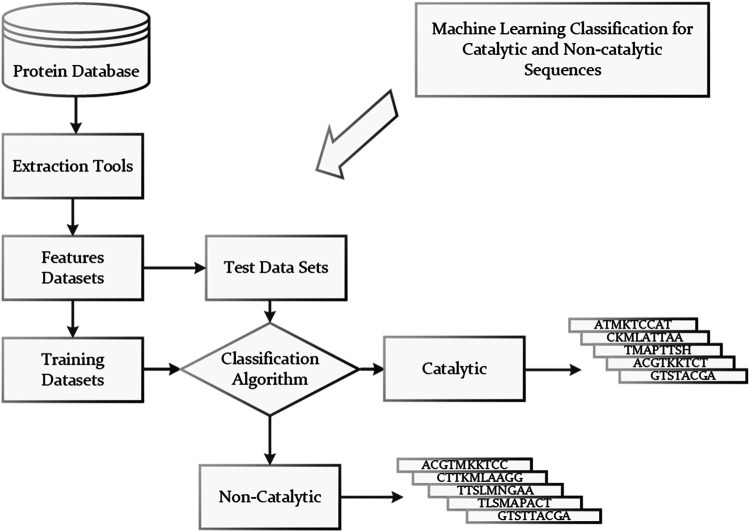
Classification of catalytic and non-catalytic protein sequences.

Now that a huge amount of data from multiple omics, such as transcriptomics, metabonomics, have been accumulated and there are many feature extracting methods (Iqbal et al., 2014; Liu et al., 2015; Du et al., 2017; Liu et al., 2017; Gao and Wu 2018; Wang et al., 2020), some researchers regarded the identification of enzyme candidates as the catalytic and non-catalytic classification problem and built models to classify protein sequences or encoding genes into either catalytic or non-catalytic by using machine learning algorithms such as support vector machine (SVM), *K*-nearest neighbors (KNN), Bayesian, and RF (Teng et al., 2010; Halperin et al., 2008; Ferrari and Mitchell 2014; Nagao et al., 2014; Amidi et al., 2017). The workflow for classifying protein sequences as catalytic and non-catalytic protein sequences is illustrated in Figure 1. The idea of such kind of methods is very simple. However, large amounts of positive (enzyme) and negative (non-enzyme) should be collected to build the models. Moreover, the predicted results can only answer whether the proteins have catalytic function, but not whether they may catalyze specific reactions.

### Evaluation of Candidates

**FIGURE 2:**
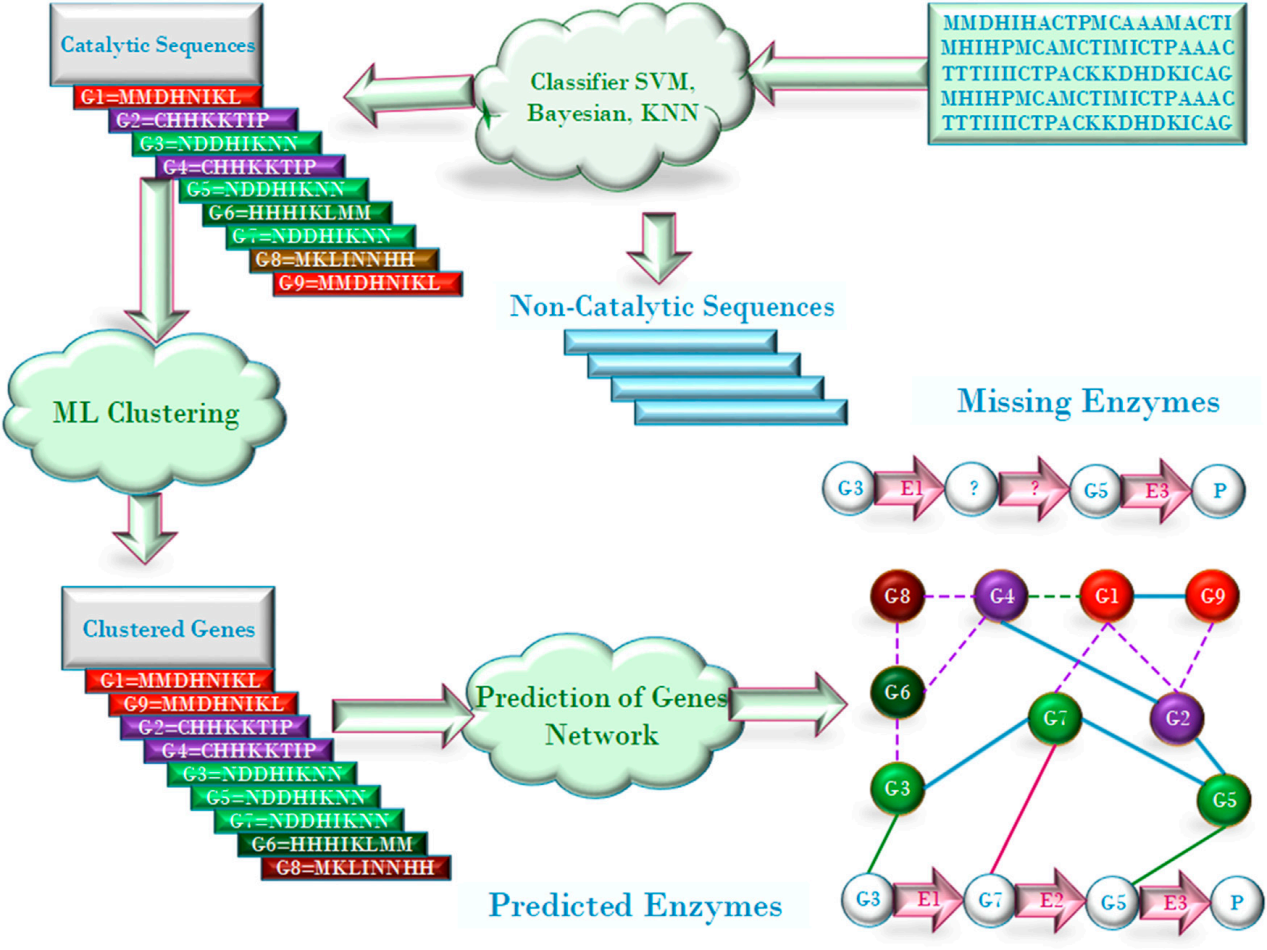
Schematic illustration of ML-based algorithms.

The purpose of evaluating candidates is to select the missing enzymes catalyzing the specific reactions from the candidates, and there have many approaches been proposed for the evaluation. For example, Green and Karp (2004) proposed Bayesian method to prioritize candidates according to the information on whether the candidate gene is located adjacent to, or in the same transcriptional unit as known enzyme-encoding genes of related metabolic function. Yamanishi et al. (2007) made the prediction of the encoding genes of missing enzymes based on the scores of the candidates and the chemical reaction information encoded in the EC number. The chemical information, including substrates, products, and chemical reactions, can be achieved from their EC numbers, using the KEGG database (Okuda et al., 2008). After the encoding genes are indicated, the functional association between genes concerning evolutionary associations and phylogenetic profiling (Rosetta and Method 2008; Nives and Dessimoz 2015; Zalguizuri et al., 2019) can be estimated and the missing enzyme can be deduced. An example of the phylogenetic profiling for filling the pathway holes is illustrated in Figure 2. Dugé de Bernonville et al. (2020) proposed several prioritization strategies, that is, by homology-based screening, by searching physical gene clusters, by random mutagenesis and by gene co-expression analysis. For the gene clustering or co-expression analysis, some algorithms have been presented to clustering gene sequences into different functional groups (Zhang et al., 2002; Zhong et al., 2005; Bustamam et al., 2017; Sharma and Ali 2017).

The problem of evaluating whether the candidate enzyme catalyzes a specific can also be regarded as the problem of predicting the interaction of substrate-enzyme-product. Chen et al. (2010) developed a KNN model for predicting substrate-enzyme-product triads. In order to measure the nearness between two triads, they defined a novel metric to weigh similarities between substrates, products, and enzymes that were calculated separately. By using their constructed benchmark date set, they got overall accuracy of 95.41%. Niu et al. (2013) also proposed KNN based model combining with mRMR-IFS (Minimum Redundancy Maximum Relevance, Incremental Feature Selection) feature selection method to predict substrate-enzyme-product triads. In order to represent each triad, they encoded substrate/product and enzyme molecules with molecular descriptors and physicochemical properties, respectively, and obtained 290 features; and then they selected 160 features that can be clustered into the ten categories. Testing on the data set that they generated based on KEGG, the model achieved the accuracy of 89.1%. Because these methods directly predict the triads, they can be used not only to predict the missing enzymes catalyzing specific reactions, but also to predict the reactions or metabolites. However, large number of labeled data is needed to promise their good performance.

## Identification of Metabolites

### Description of the Problem

The metabolites are small molecules which are used in, or created by the chemical reactions occurring in every cell of living organisms. The reactants, intermediates, and products in a metabolic pathway are all called metabolites. Interpreting biochemical characteristics of the metabolites is an essential part of the metabolomics to extend the knowledge of biological systems. It is also the key to the development of many applications in areas such as biotechnology, biomedicine or pharmaceuticals (Nguyen et al., 2019). The identification of the metabolites remains a challenging task in metabolomics with a huge number of potentially interesting but unknown metabolites. Nuclear magnetic resonance (NMR) spectroscopy and mass spectrometry (MS) hyphenated with separation techniques such as liquid chromatography (LC), gas chromatography (GC) and capillary electrophoresis (CE) are the most frequently used techniques to collect large amounts of data on complex biological mixtures or matrices (Wachsmuth et al., 2013). They typically yield complicated spectra or feature-rich chromatograms containing thousands of unknown or unidentified peaks. NMR has the disadvantage that it requires abundant and pure samples, yielding low sensitivity. By contrast, MS is more sensitive and specific, requiring fewer amount of samples (Nguyen et al., 2019). Therefore, most methods for identifying metabolites are based on the MS (Yi et al., 2018). The identification of small molecules from MS data remains a major challenge.

### Identification of Metabolites

**FIGURE 3:**
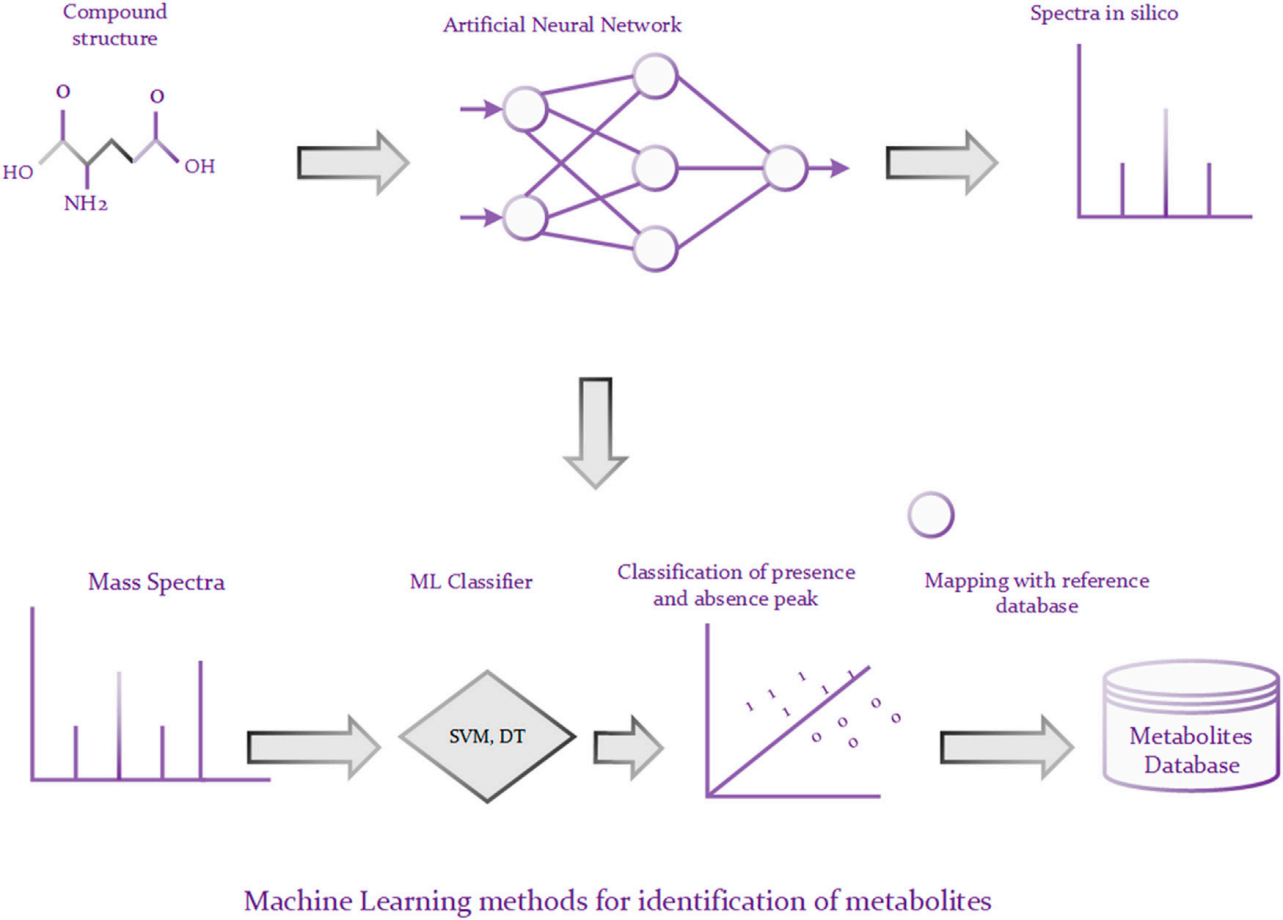
Prediction of metabolites using ML techniques.

A traditional approach to identifying metabolites is to compare a query MS or MS/MS spectrum of an unknown compound against a database, such as METLIN (Smith et al., 2005), of a number of reference MS or MS/MS spectra. The candidate molecules from the database are ranked based on the similarity of their spectra and the query spectrum and the best matching candidates are returned. Though such methods are reliable, they are only helpful for those unknown metabolites that have reference spectra in the database (Hufsky et al., 2014). Unfortunately, the reference database is often incomplete in reality, leading to unreliable matching results if the reference spectrum of the targeted compound is not contained in the database (Nguyen et al., 2019). To alleviate above problem, a lot of machine learning based approaches have been proposed to predict metabolites *via* learning the spectra patterns of the known compounds. For example, Kangas et al. (2012) developed an algorithm based on Monte Carlo simulations for identifying metabolites. The algorithm has two phases, illustrated in Figure 3. In the first phase, it predicts bond cleavage energies from which cleavage rates can be calculated based on the ANN (Artificial Neural Network). In the second phase, it generates in silico tandem mass spectra from molecular structures and uses these spectra for the identification. There are roughly two schemas for machine learning methods (Nguyen et al., 2019). Some methods rely on predicting molecular fingerprints from MS/MS data and finding the most similar fingerprint from the molecular structure database (Dührkop et al., 2015; Brouard et al., 2016; Brouard et al., 2019). And the other methods call for predicting MS/MS spectra for a set of candidate molecular structures and choosing the most similar predicted MS/MS spectrum to the observed MS/MS spectrum (Allen et al., 2014; Shen et al., 2014; Djoumbou-Feunang et al., 2019). Those approaches have achieved good identification performance. However, they are highly sensitive and generally cannot model non-linear relationship. It is known that deep learning architecture can be used to build internal representation of large non-linear data, which may lead to superior predictive performance compared to traditional machine learning algorithms. For instance, graph convolution neural network can be directly used to process the graph structure of small molecules, where nodes represent the atoms and edges stand for the bonds between atoms. Moreover, different variants of graph convolution neural network, such as spatial graph convolution networks and spectral graph convolution networks, can be used to optimize the predictive performance.

## Prediction of Reactions

### Description of the Problem

With the great developments in metabolomics and synthetic biology, on one hand a large amount of data related on metabolic pathways has been generated and been organized in several databases, such as KEGG (Okuda et al., 2008), BioCyc (Karp et al., 2019), and MetaCyc (Karp 2002a; Caspi 2006). On the other hand, it is assumed that a large number of metabolic pathways remain unknown, and many reactions are still missing even in known pathways. What’s more, there is an increasing number of compounds that are known to be present in living organisms but whose synthetic/degradation pathways are unknown. The missing of one or more reactions may result that the pathways from an initial compound to the desired target in an organism are incomplete. Therefore, it is necessary identify such missing reactions during the reconstruction of metabolic pathways. In the field of biosynthesis, finding the potential connection betweeen two known pathways by introducing a novel reaction may lead to a new pathway to the desired product.

### Prediction of Reactions

Reaction prediction remains a challenging task for investigating metabolic pathways due to resonance structure and specific products that can be redundant and problematic. However, recent machine learning developments have alleviated this problem, resulting in additional performance (Cuperlovic-Culf, 2018). According to whether compounds or pairs of compounds are used in modeling, there are two kinds of roadmaps for reaction prediction: focusing compounds (Kotera et al., 2008; Wei et al., 2016) and focusing compound pairs (Mu et al., 2011; Kotera et al., 2013; Fooshee et al., 2018).

**FIGURE 4:**
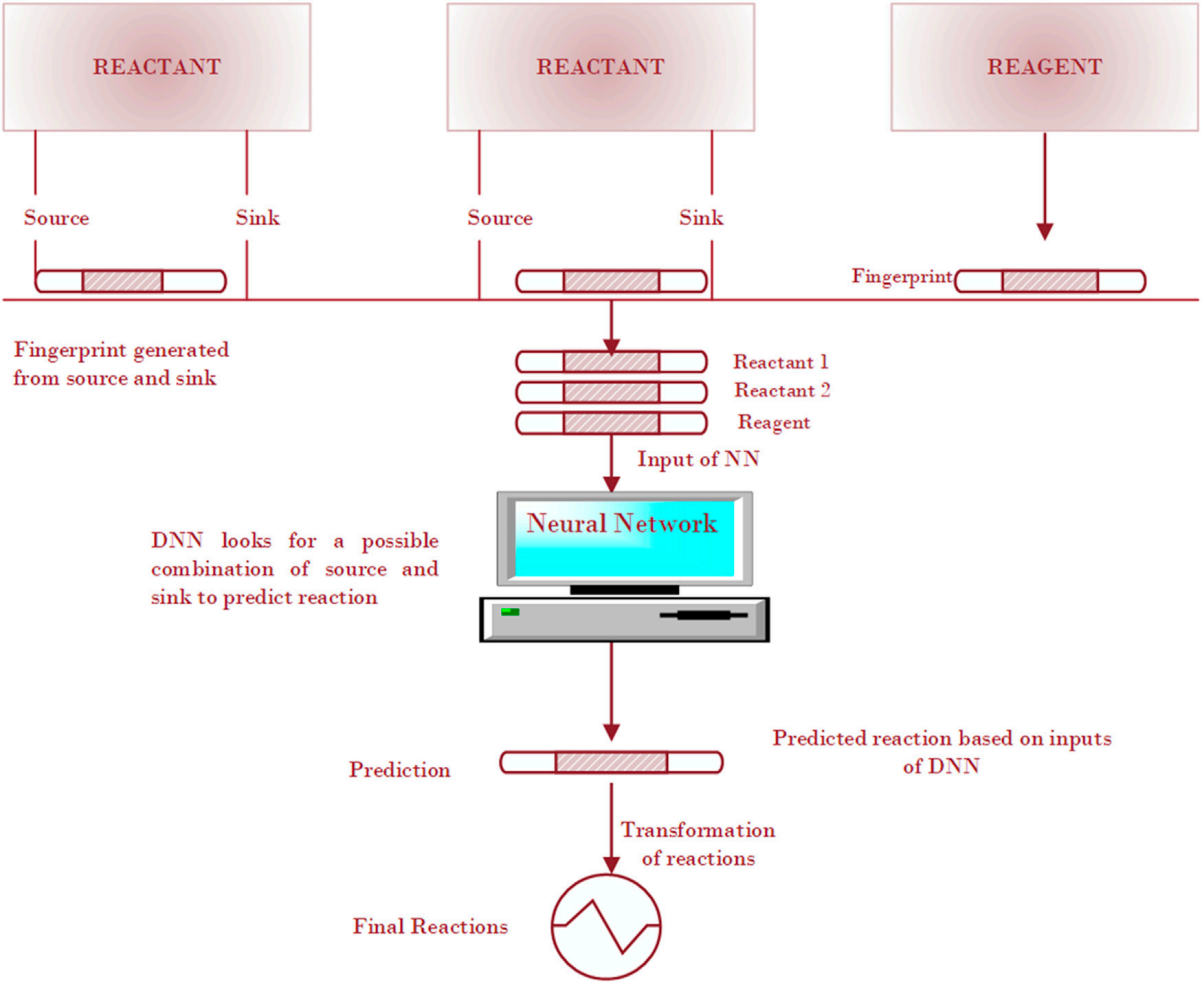
Schematic illustration of a deep neural network method for the prediction of a reaction.

The compound-focused methods identify products or precursors for given compounds and then generate the plausible reactions. For example, Kotera et al. (2008) presented a substructure-based approach to identify possible products and/or precursors for a given compound and to generate a plausible reaction. By using the RF methods, they searched compounds that were structurally related to the target compound, and the structural differences were then checked to determine which of these has the potential to be a product (or precursor) of the target compound in an enzyme-catalyzed reaction. Wei et al. (2016) followed the similar roadmap. Given a set of reagents and reactants, they first built a neural network to predict the reaction type based on a reaction fingerprinting method, and then they used SMARTS (SMiles ARbitrary Target Specification) transformation to predict the likely product from reactants. The neural network workflow starts with reactant and reagent molecules and enumerates all possible electron sources and sinks within the input molecules, based on the atom and bond descriptors, shown in Figure 4. The fingerprinting approach is based on a specific pattern of the molecules, searching occurs all around the molecular structure to detect the presence and absence of the specific pattern in the molecule. The fingerprints for concatenated reactants and reagents become the input for the neural network to predict possible reaction types.

The compound pair-focused methods aim for predicting whether a given compound-compound pair is possibly reactive or not. For instance, Mu et al. (2011) built SVM classifiers to discriminate between functional groups that are reactive and non-reactive. To train the classifiers, they collected positive and negative examples from the KEGG database for each SMARTS-defined substructure, and used atomic properties of atoms in putative reaction centers and molecular properties as features. Kotera et al. (2013) applied a sparsity-induced classifier and SVM to learn whether a compound-compound pair is possibly converted to each other by enzymatic reactions. In order to represent the samples, they defined feature vectors representing the chemical transformation patterns of compound-compound pairs in enzymatic reactions by using chemical fingerprints. Recently, Fooshee et al. (2018) presented a deep learing based reaction prediction method that operated at the level of elementary reactions. Each elementary step involves the movement of electrons from an electron source to an electron sink, and all elementary reactions can be chained together to yield the complex global reaction.

## Conclusion

The prediction and construction of synthetic metabolic pathways is a significant challenge in bioinformatics. Machine Learning techniques play important roles in constructing and understanding metabolic pathways and their subparts. This mini review provided the outline of the applications of machine learning approaches for prediction and reconstruction of metabolic pathways. Some related issues needed to be addressed were also discussed. Moreover, some machine learning based methods for the identification of missing enzymes, metabolites, or reactions were introduced in this paper. This review complements the existing review work and can provide more comprehensive knowledge for machine learning algorithms in the prediction and reconstruction of the metabolic pathways.

## References

[B1] Allen F. Pon A. Wilson M. Greiner R. Wishart D. (2014). CFM-ID: A Web Server for Annotation, Spectrum Prediction and Metabolite Identification from Tandem Mass Spectra. Nucleic Acids Res. 42, W94–W99. 10.1093/nar/gku436 24895432 PMC4086103

[B2] Amidi S. Amidi A. Vlachakis D. Paragios N. Zacharaki E. I. (2017). Automatic Single- and Multi-Label Enzymatic Function Prediction by Machine Learning. PeerJ 5 (3), e3095–16. 10.7717/peerj.3095 28367366 PMC5374972

[B3] Arabzadeh M. Saheb Zamani M. Sedighi M. Marashi S.-A. (2018). A Graph-Based Approach to Analyze Flux-Balanced Pathways in Metabolic Networks. BioSystems 165, 40–51. 10.1016/j.biosystems.2017.12.001 29337084

[B4] Aziz R. K. Bartels D. Best A. A. DeJongh M. Disz T. Edwards R. A. (2008). The RAST Server: Rapid Annotations Using Subsystems Technology. BMC Genomics 9, 75–15. 10.1186/1471-2164-9-75 18261238 PMC2265698

[B5] Bagheri M. Marashi S.-A. Amoozegar M. A. (2019). A Genome-Scale Metabolic Network Reconstruction of Extremely Halophilic Bacterium Salinibacter Ruber. PLoS One 14 (5), e0216336–17. 10.1371/journal.pone.0216336 31071110 PMC6508672

[B6] Baranwal M. Magner A. Elvati P. Saldinger J. Violi A. Hero A. O. (2019). A Deep Learning Architecture for Metabolic Pathway Prediction. Bioinformatics 36 (8), 2547–2553. 10.1093/bioinformatics/btz954 31879763

[B7] Besemer J. (2001). GeneMarkS: a Self-Training Method for Prediction of Gene Starts in Microbial Genomes. Implications for Finding Sequence Motifs in Regulatory Regions. Nucleic Acids Res. 29 (12), 2607–2618. 10.1093/nar/29.12.2607 11410670 PMC55746

[B8] Brouard C. Bassé A. d’Alché-Buc F. Rousu J. (2019). Improved Small Molecule Identification through Learning Combinations of Kernel Regression Models. Metabolites 9, 160. 10.3390/metabo9080160 PMC6724104 31374904

[B9] Brouard C. Shen H. Dührkop K. d'Alché-Buc F. Böcker S. Rousu J. (2016). Fast Metabolite Identification with Input Output Kernel Regression. Bioinformatics 32, i28–i36. 10.1093/bioinformatics/btw246 27307628 PMC4908330

[B10] Bustamam A. Tasman H. Yuniarti N. Frisca Mursidah I. (2017). Application of K-Means Clustering Algorithm in Grouping the DNA Sequences of Hepatitis B Virus (HBV). AIP Conf. Proc. 1862:030134. 10.1063/1.4991238

[B11] Caspi R. Billington R. Fulcher C. A. Keseler I. M. Kothari A. Krummenacker M. (2018). The MetaCyc Database of Metabolic Pathways and Enzymes. Nucleic Acids Res. 46 (D1), D633–D639. 10.1093/nar/gkx935 29059334 PMC5753197

[B12] Caspi R. Foerster H. Fulcher C. A. Kaipa P. Krummenacker M. Latendresse M. (2008). The MetaCyc Database of Metabolic Pathways and Enzymes and the BioCyc Collection of Pathway/genome Databases. Nucleic Acids Res. 36, D623–D631. 10.1093/nar/gkm900 17965431 PMC2238876

[B13] Caspi R. (2006). MetaCyc: a Multiorganism Database of Metabolic Pathways and Enzymes. Nucleic Acids Res. 34, D511–D516. 10.1093/nar/gkj128 16381923 PMC1347490

[B14] Chen L. Feng K.-Y. Cai Y.-D. Chou K.-C. Li H.-P. (2010). Predicting the Network of Substrate-Enzyme-Product Triads by Combining Compound Similarity and Functional Domain Composition. BMC Bioinformatics 11, 293. 10.1186/1471-2105-11-293 20513238 PMC3098070

[B15] Connor W. C. Rogers L Green W. H, (2017). “Computer-Assisted Retrosynthesis Based on Molecular Similarity”, ACS Cent. Sci., 3, 1237–1245. 29296663 10.1021/acscentsci.7b00355 PMC5746854

[B16] Cuperlovic-Culf M. (2018). Machine Learning Methods for Analysis of Metabolic Data and Metabolic Pathway Modeling, Metabolites, 8, 4. 10.3390/metabo8010004 PMC5875994 29324649

[B18] Delépine B. Duigou T. Carbonell P. Faulon J.-L. (2018). RetroPath2.0: A Retrosynthesis Workflow for Metabolic Engineers. Metab. Eng. 45, 158–170. 10.1016/j.ymben.2017.12.002 29233745

[B19] Djoumbou-Feunang Y. Pon A. Karu N. Zheng J. Li C. Arndt D. (2019). CFM-ID 3.0: Significantly Improved ESI-MS/MS Prediction and Compound Identification. Metabolites 9, 72. 10.3390/metabo9040072 PMC6523630 31013937

[B20] Du P.-F. Zhao W. Miao Y.-Y. Wei L.-Y. Wang L. (2017). Ultrapse: A Universal and Extensible Software Platform for Representing Biological Sequences. Ijms 18, 2400–2411. 10.3390/ijms18112400 PMC5713368 29135934

[B21] Dugé de Bernonville T. Papon N. Clastre M. O'Connor S. E. Courdavault V. (2020). Identifying Missing Biosynthesis Enzymes of Plant Natural Products. Trends Pharmacol. Sci. 41 (3), 142–146. 10.1016/j.tips.2019.12.006 31980250

[B22] Dührkop K. Shen H. Meusel M. Rousu J. Böcker S. (2015). Searching Molecular Structure Databases with Tandem Mass Spectra Using CSI:FingerID. Proc. Natl. Acad. Sci. USA 112, 12580–12585. 10.1073/pnas.1509788112 26392543 PMC4611636

[B23] Ebenhöh O. Heinrich R. (2001). Evolutionary Optimization of Metabolic Pathways. Theoretical Reconstruction of the Stoichiometry of ATP and NADH Producing Systems. Bull. Math. Biol. 63 (1), 21–55. 10.1006/bulm.2000.0197 11146883

[B24] Faust K. Croes D. van Helden J. (2011). Prediction of Metabolic Pathways from Genome-Scale Metabolic Networks. BioSystems 105 (2), 109–121. 10.1016/j.biosystems.2011.05.004 21645586

[B25] Ferrari L. Mitchell J. B. O. (2014). From Sequence to Enzyme Mechanism Using Multi-Label Machine Learning. BMC Bioinformatics 15 (1), 1–13. 10.1186/1471-2105-15-150 24885296 PMC4229970

[B26] Fooshee D. Mood A. Gutman E. Tavakoli M. Urban G. Liu F. (2018). Deep Learning for Chemical Reaction Prediction. Mol. Syst. Des. Eng. 3 (3), 442–452. 10.1039/c7me00107j

[B27] Friedman N. (2004). Inferring Cellular Networks Using Probabilistic Graphical Models. Science 303, 799–805. 10.1126/science.1094068 14764868

[B28] Gao C. F. Wu X. Y. (2018). Feature Extraction Method for Proteins Based on Markov Tripeptide by Compressive Sensing. BMC Bioinformatics 19 (1), 1–10. 10.1186/s12859-018-2235-x 29914376 PMC6006778

[B29] Gerlee P. Lizana L. Sneppen K. (2009). Pathway Identification by Network Pruning in the Metabolic Network of *Escherichia coli* . Bioinformatics 25 (24), 3282–3288. 10.1093/bioinformatics/btp575 19808881

[B31] Green M. L. Karp P. D. (2004). A Bayesian Method for Identifying Missing Enzymes in Predicted Metabolic Pathway Databases. BMC Bioinformatics 5, 76. 10.1186/1471-2105-5-76 15189570 PMC446185

[B100] Gurkun B. (2012). “Identifying Gene Interaction Networks,” in Statistical Human Genetics Methods and Protocols. Editors Robert C. E. Jaya M. S. Sun S. (Totowa, NY: Humana Press), 483–494. 10.1007/978-1-61779-555-8_26

[B32] Halperin I. Glazer D. S. Wu S. Altman R. B. (2008). The FEATURE Framework for Protein Function Annotation: Modeling New Functions, Improving Performance, and Extending to Novel Applications. BMC Genomics 9 (2), S2–S14. 10.1186/1471-2164-9-S2-S2 PMC2559884 18831785

[B99] Herrgård M. J. Swainston N. Dobson P. Dunn W. B. Arga K. Y. Arvas M. (2008). A Consensus Yeast Metabolic Network Reconstruction Obtained from a Community Approach to Systems Biology. Nat. Biotechnol. 26 (10), 1155–1160. 10.1038/nbt1492 18846089 PMC4018421

[B33] Hufsky F. Scheubert K. Böcker S. (2014). Computational Mass Spectrometry for Small-Molecule Fragmentation. Trac Trends Anal. Chem. 53, 41–48. 10.1016/j.trac.2013.09.008

[B34] Iqbal M. J. Faye I. Samir B. B. Md Said A. (2014). Efficient Feature Selection and Classification of Protein Sequence Data in Bioinformatics. Scientific World J. 2014, 1–12. 10.1155/2014/173869 PMC4089199 25045727

[B35] Jansen R. Yu H Emili A Kluger Y Greenbaum D Chung S (2003). A Bayesian Networks Approach for Predicting Protein-Protein Interactions from Genomic Data. Science 302, 449–453. 10.1126/science.1087361 14564010

[B36] Jeanne G. Tebbani S. Goelzer A. Fromion V. Dumur D. (2016). “Modelling and Optimization of Metabolic Pathways in Bacteria,” in Int. Conf. Syst. Theory, Control Comput. ICSTCC 2016 - Jt. Conf. SINTES 20, Sinaia, Romania, 13-15 Oct. 2016 (IEEE), 312–317. 10.1109/ICSTCC.2016.7790684

[B37] Jeske L. Placzek S. Schomburg I. Chang A. Schomburg D. (2019). BRENDA in 2019: A European ELIXIR Core Data Resource. Nucleic Acids Res. 47 (D1), D542–D549. 10.1093/nar/gky1048 30395242 PMC6323942

[B38] Jia Y. Zhao R. Chen L. (2020). Similarity-Based Machine Learning Model for Predicting the Metabolic Pathways of Compounds. IEEE Access 8, 130687–130696. 10.1109/access.2020.3009439

[B39] Küken A. Nikoloski Z. (2019). “Computational Approaches to Design and Test Plant Synthetic Metabolic Pathways,” Plant Physiol. 179 (3), 894–906. 10.1104/pp.18.01273 30647083 PMC6393797

[B40] Kanehisa M. Sato Y. Furumichi M. Morishima K. Tanabe M. (2019). New Approach for Understanding Genome Variations in KEGG. Nucleic Acids Res. 47 (D1), D590–D595. 10.1093/nar/gky962 30321428 PMC6324070

[B41] Kanehisa M. Sato Y. Morishima K. (2016). BlastKOALA and GhostKOALA: KEGG Tools for Functional Characterization of Genome and Metagenome Sequences. J. Mol. Biol. 428 (4), 726–731. 10.1016/j.jmb.2015.11.006 26585406

[B42] Kangas L. J. Metz T. O. Isaac G. Schrom B. T. Ginovska-Pangovska B. Wang L. (2012). In Silico identification Software (ISIS): A Machine Learning Approach to Tandem Mass Spectral Identification of Lipids. Bioinformatics 28 (13), 1705–1713. 10.1093/bioinformatics/bts194 22592377 PMC3381961

[B43] Karp P. D. Billington R. Caspi R. Fulcher C. A. Latendresse M. Kothari A. (2019). The BioCyc Collection of Microbial Genomes and Metabolic Pathways. Brief. Bioinform. 20 (4), 1085–1093. 10.1093/bib/bbx085 29447345 PMC6781571

[B44] Karp P. D. Krummenacker M. Paley S. Wagg J. (1999). Integrated Pathway-Genome Databases and Their Role in Drug Discovery. Trends Biotechnol. 17 (7), 275–281. 10.1016/s0167-7799(99)01316-5 10370234

[B45] Karp P. D. (2002a). The EcoCyc Database. Nucleic Acids Res. 30 (1), 56–58. 10.1093/nar/30.1.56 11752253 PMC99147

[B46] Karp P. D. (2002b). The MetaCyc Database. Nucleic Acids Res. 30 (1), 59–61. 10.1093/nar/30.1.59 11752254 PMC99148

[B47] Kharchenko P. Chen L. Freund Y. Vitkup D. Church G. M. (2006). Identifying Metabolic Enzymes with Multiple Types of Association Evidence. BMC Bioinformatics 7, 177. 10.1186/1471-2105-7-177 16571130 PMC1450304

[B48] Kim G. B. Kim W. J. Kim H. U. Lee S. Y. (2020). Machine Learning Applications in Systems Metabolic Engineering. Curr. Opin. Biotechnol. 64, 1–9. 10.1016/j.copbio.2019.08.010 31580992

[B49] Koch M. Duigou T. Faulon J.-L. (2020). “Reinforcement Learning for Bioretrosynthesis”, ACS Synth. Biol., 9, 157–168. 10.1021/acssynbio.9b00447 31841626

[B50] Kotera M. McDonald A. G. Boyce S. Tipton K. F. (2008). Eliciting Possible Reaction Equations and Metabolic Pathways Involving Orphan Metabolites. J. Chem. Inf. Model. 48 (12), 2335–2349. 10.1021/ci800213g 19053521

[B51] Kotera M. Tabei Y. Yamanishi Y. Tokimatsu T. Goto S. (2013). Supervised De Novo Reconstruction of Metabolic Pathways from Metabolome-Scale Compound Sets. Bioinformatics 29 (13), i135–i144. 10.1093/bioinformatics/btt244 23812977 PMC3694648

[B52] Koza J. R. Mydlowec W. Lanza G. Yu J. Keane M. A. (2001). Reverse Engineering of Metabolic Pathways from Observed Data Using Genetic Programming. Pac. Symp. Biocomput 2001, 434–445. 10.1142/9789814447362_0043 11262962

[B56] Lin G.-M. Warden-Rothman R. Voigt C. A. (2019). Retrosynthetic Design of Metabolic Pathways to Chemicals Not Found in Nature. Curr. Opin. Syst. Biol. 14, 82–107. 10.1016/j.coisb.2019.04.004

[B57] Liu B. Liu F. Wang X. Chen J. Fang L. Chou K.-C. (2015). Pse-in-One: A Web Server for Generating Various Modes of Pseudo Components of DNA, RNA, and Protein Sequences. Nucleic Acids Res. 43, W65–W71. 10.1093/nar/gkv458 25958395 PMC4489303

[B58] Liu B. Wu H. Zhang D. Wang X. Chou K.-C. (2017). Pse-Analysis: a python Package for DNA/RNA and Protein/peptide Sequence Analysis Based on Pseudo Components and Kernel Methods. Oncotarget 8 (8), 13338–13343. 10.18632/oncotarget.14524 28076851 PMC5355101

[B59] Lombardot T. Morgat A. Axelsen K. B. Aimo L. Hyka-Nouspikel N. Niknejad A. (2019). Updates in Rhea: SPARQLing Biochemical Reaction Data, Nucleic Acids Res. 47, D596–D600. 10.1093/nar/gky876 30272209 PMC6324061

[B60] Mascher M. Schreiber M. Scholz U. Graner A. Reif J. C. Stein N. (2019). Genebank Genomics Bridges the gap between the Conservation of Crop Diversity and Plant Breeding, Nat. Genet. 51. 1076–1081. 10.1038/s41588-019-0443-6 31253974

[B61] Moriya Y. Itoh M. Okuda S. Yoshizawa A. C. Kanehisa M. (2007). KAAS: An Automatic Genome Annotation and Pathway Reconstruction Server. Nucleic Acids Res. 35, W182–W185. 10.1093/nar/gkm321 17526522 PMC1933193

[B63] Mu F. Unkefer C. J. Unkefer P. J. Hlavacek W. S. (2011). Prediction of Metabolic Reactions Based on Atomic and Molecular Properties of Small-Molecule Compounds. Bioinformatics 27 (11), 1537–1545. 10.1093/bioinformatics/btr177 21478194 PMC3102224

[B64] Nagao C. Nagano N. Mizuguchi K. (2014). Prediction of Detailed Enzyme Functions and Identification of Specificity Determining Residues by Random Forests. PLoS One 9 (1), 1–12. 10.1371/journal.pone.0084623 PMC3885575 24416252

[B65] Nguyen D. H. Nguyen C. H. Mamitsuka H. (2019). Recent Advances and Prospects of Computational Methods for Metabolite Identification: A Review with Emphasis on Machine Learning Approaches. Brief. Bioinform. 20 (6), 2028–2043. 10.1093/bib/bby066 30099485 PMC6954430

[B66] Niu B. Huang G. Zheng L. Wang X. Chen F. Zhang Y. (2013). Prediction of Substrate-Enzyme-Product Interaction Based on Molecular Descriptors and Physicochemical Properties. Biomed. Res. Int. 2013, 1–7. 10.1155/2013/674215 PMC3881445 24455714

[B67] Nives Š. Dessimoz C. (2015). Phylogenetic Profiling : How Much Input Data Is Enough? plos one 10, e0114701. 10.1371/journal.pone.0114701 25679783 PMC4332489

[B68] Ogata H. Goto S. Fujibuchi W. Kanehisa M. (1998). Computation with the KEGG Pathway Database. BioSystems 47 (1), 119–128. 10.1016/S0303-2647(98)00017-3 9715755

[B69] Ogata H. Goto S. Sato K. Fujibuchi W. Bono H. Kanehisa M. (1999). KEGG: Kyoto Encyclopedia of Genes and Genomes. Nucleic Acids Res. 27 (1), 29–34. 10.1093/nar/27.1.29 9847135 PMC148090

[B70] Okuda S. Yamada T. Hamajima M. Itoh M. Katayama T. Bork P. (2008). KEGG Atlas Mapping for Global Analysis of Metabolic Pathways. Nucleic Acids Res. 36, 423–426. 10.1093/nar/gkn282 18477636 PMC2447737

[B71] Overbeek R. (2000). WIT: Integrated System for High-Throughput Genome Sequence Analysis and Metabolic Reconstruction. Nucleic Acids Res. 28 (1), 123–125. 10.1093/nar/28.1.123 10592199 PMC102471

[B72] Paley S. M. Karp P. D. (2002). Predictions for *Helicobacter pylori* . Bioinformatics 18 (5), 715–724. 10.1093/bioinformatics/18.5.715 12050068

[B73] Planes F. J. Beasley J. E. (2009). An Optimization Model for Metabolic Pathways. Bioinformatics 25 (20), 2723–2729. 10.1093/bioinformatics/btp441 19620100

[B74] Planson A. G. Carbonell P Grigoras L Faulon J. L, (2012). A Retrosynthetic Biology Approach to Therapeutics: from conception to Delivery. Curr. Opin. Biotechnol. 23, 948–956. 10.1016/j.copbio.2012.03.009 22475981

[B75] Qi Q. Li J. Cheng J. (2014). Reconstruction of Metabolic Pathways by Combining Probabilistic Graphical Model-Based and Knowledge-Based Methods. BMC Proc. 8 (6), 1–10. 10.1186/1753-6561-8-S6-S5 PMC4202177 25374614

[B76] Roche-Lima A. (2016). Implementation and Comparison of Kernel-Based Learning Methods to Predict Metabolic Networks. Netw. Model. Anal. Heal. Inform. Bioinforma. 5 (1), 1–7. 10.1007/s13721-016-0134-5 PMC4947111 27471658

[B77] Rosetta T. Method S. (2008). Chapter 7 the Rosetta Stone Method. Methods Mol. Biol. 453, 169–180. 10.1007/978-1-60327-429-610.1007/978-1-60327-429-6_7 18712302

[B78] Schmidt M. D. Vallabhajosyula R. R. Jenkins J. W. Hood J. E. Soni A. S. Wikswo J. P. (2011). Automated Refinement and Inference of Analytical Models for Metabolic Networks. Phys. Biol. 8 (5), 055011. 10.1088/1478-3975/8/5/055011 21832805 PMC4109817

[B79] Schomburg I. (2002). BRENDA, Enzyme Data and Metabolic Information. Nucleic Acids Res. 30 (1), 47–49. 10.1093/nar/30.1.47 11752250 PMC99121

[B80] Sharma A. Ali H. H. (2017). Analysis of Clustering Algorithms in Biological Networks. Proc. - 2017 IEEE Int. Conf. Bioinforma. Biomed. BIBM 2017, 2303–2305. 10.1109/BIBM.2017.8218036

[B81] Shen H. Dührkop K. Böcker S. Rousu J. (2014). Metabolite Identification through Multiple Kernel Learning on Fragmentation Trees. Bioinformatics 30 (12), 157–164. 10.1093/bioinformatics/btu275 24931979 PMC4058957

[B82] Sithambranathan M. Kasim S. Hassan M. Z. Syafi N. A. (2020). Clustering of Genes Skin' S Cancer, Intelligence Comput. 1, 1–9. 10.18517/ijods.1.1.51-56.2020

[B83] Smith C. A. Maile O. G Want E. J Qin C Trauge S. A Brandon T. R (2005). METLIN: A Metabolite Mass Spectral Database. Ther. Drug Monit. 27 (6), 747–751. 10.1097/01.ftd.0000179845.53213.39 16404815

[B84] Teng S. Srivastava A. K. Wang L. (2010). Sequence Feature-Based Prediction of Protein Stability Changes upon Amino Acid Substitutions. BMC Genomics 11 (2), S5. 10.1186/1471-2164-11-S2-S5 PMC2975416 21047386

[B86] Viswanathan G. A. Seto J. Patil S. Nudelman G. Sealfon S. C. (2008). Getting Started in Biological Pathway Construction and Analysis. Plos Comput. Biol. 4 (2), 16. 10.1371/journal.pcbi.0040016 PMC2323403 18463709

[B87] Wachsmuth C. J. Vogl F. C. Oefner P. J. Dettmer K. (2013). Gas Chromatographic Techniques in Metabolomics. RSC Chromatogr. Monogr. Chromatogr. Methods Metabolomics. 87–113. 10.1039/9781849737272-00087

[B88] Wang J. Du P. F. Xue X. Y. Li G. P. Zhou Y. K. Zhao W. (2020). VisFeature: A Stand-Alone Program for Visualizing and Analyzing Statistical Features of Biological Sequences. Bioinformatics 36 (4), 1277–1278. 10.1093/bioinformatics/btz689 31504195

[B89] Wang L. Dash S. Ng C. Y. Maranas C. D. (2017). A Review of Computational Tools for Design and Reconstruction of Metabolic Pathways. Synth. Syst. Biotechnol. 2 (4), 243–252. 10.1016/j.synbio.2017.11.002 29552648 PMC5851934

[B90] Wei J. N. Duvenaud D. Aspuru-Guzik A. (2016). Neural Networks for the Prediction of Organic Chemistry Reactions. ACS Cent. Sci. 2 (10), 725–732. 10.1021/acscentsci.6b00219 27800555 PMC5084081

[B91] Werhli A. V. Grzegorczyk M. Husmeier D. (2006). Comparative Evaluation of Reverse Engineering Gene Regulatory Networks with Relevance Networks, Graphical Gaussian Models and Bayesian Networks. Bioinformatics 22, 2523–2531. 10.1093/bioinformatics/btl391 16844710

[B93] Yamanishi Y. Mihara H. Osaki M. Muramatsu H. Esaki N. Sato T. (2007). Prediction of Missing Enzyme Genes in a Bacterial Metabolic Network: Reconstruction of the Lysine-Degradation Pathway of *Pseudomonas aeruginosa* . FEBS J. 274 (9), 2262–2273. 10.1111/j.1742-4658.2007.05763.x 17388807

[B94] Yi J. J. Park K. Kim W. J. Rhee J. K. Son W. S. (2018). Spectroscopic Methods to Analyze Drug Metabolites. Arch. Pharmacal Res. 41 (4), 355–371. 10.1007/s12272-018-1010-x 29524156

[B95] Zalguizuri A. Caetano-Anollés G. Lepek V. C. (2019). Phylogenetic Profiling, an Untapped Resource for the Prediction of Secreted Proteins and its Complementation with Sequence-Based Classifiers in Bacterial Type III, IV and VI Secretion Systems. Brief. Bioinform. 20 (4), 1395–1402. 10.1093/bib/bby009 29394318

[B96] Zhang S. Liao L. Tomb J.-F. Wang J. T. L. (2002). Clustering and Classifying Enzymes in Metabolic Pathways : Some Preliminary Results. BIOKDD’02 Proceedings of the 2nd International Conference on Data Mining in Bioinformatics, Berlin, Heidelberg: Springer-Verlag, 23 July 2002 19–24. Available at: http://dl.acm.org/citation.cfm?id=3012339.

[B97] Zhao Y. Chen M-H. Pei B. Rowe D. Shin D. G. Xie W. (2012). A Bayesian Approach to Pathway Analysis by Integrating Gene-Gene Functional Directions and Microarray Data. Stat. Biosciences 4, 105–131. 10.1007/s12561-011-9046-1 PMC3592971 23482678

[B98] Zhong W. Altun G. Harrison R. Tai P. C. Pan Y. (2005). Improved K-Means Clustering Algorithm for Exploring Local Protein Sequence Motifs Representing Common Structural Property. IEEE Trans. Nanobioscience 4 (3), 255–265. 10.1109/TNB.2005.853667 16220690

